# Combined Efficacy of CXCL5, STC2, and CHI3L1 in the Diagnosis of Colorectal Cancer

**DOI:** 10.1155/2022/7271514

**Published:** 2022-05-20

**Authors:** Hui Li, Xiaoping Zhou, Haining Zhang, Jifeng Jiang, Hu Fu, Fang Wang

**Affiliations:** ^1^Department of Laboratory Medicine, Chengdu First People's Hospital, Chengdu, Sichuan 610041, China; ^2^Department of Laboratory Medicine, The First Affiliated Hospital of Xi'an Jiaotong University, Xi'an, Shaanxi 710061, China

## Abstract

**Objective:**

To improve the diagnostic capacity of serum biomarkers for colorectal cancer (CRC), we introduced three novel indicators, namely, the C-X-C motif chemokine ligand 5 (CXCL5), stanniocalcin 2 (STC2), and chitinase 3 like 1 (CHI3L1) and assessed their performances in the detection of CRC.

**Methods:**

A total of 887 serum samples (153 health, 342 polyps, and 392 CRCs) were collected. Concentrations of CXCL5, STC2, and CHI3L1 were measured by the ELISA. CEA and CA199 were determined by electrochemiluminescence. Binary logistic regression was used to build the combination model. ROC analysis was used to evaluate the performance of biomarkers alone or in combination.

**Results:**

Model_2 that based on CXCL5, STC2, and CHI3L1 was the best approach in discriminating CRC from non-CRC controls (AUC, 0.943 (0.922–0.960); sensitivity, 0.848; specificity, 0.917; and accuracy, 0.887 in the training cohort and 0.959 (95% CI 0.927–0.980), 0.878, 0.917, and 0.900 in the testing cohort, respectively). In the detection of early CRC, Model_2 revealed AUC, sensitivity, specificity, and accuracy of 0.925 (0.897–0.947), 0.793, 0.917, and 0.886 in the training cohort and those of 0.926 (0.979–0.959), 0.786, 0.931, and 0.898 in the testing cohort. Furthermore, Model_2 exhibited an excellent diagnostic performance in CEA-negative cases (0.938 (0.913–0.957), 0.826, 0.917, and 0.888 in the training cohort and 0.961 (0.925–0.983), 0.887, 0.931, and 0.918 in the testing cohort). As used alone, STC2 achieved the capacities that is second only to that of Model_2 (0.866 (0.837–0.892), 0.859, 0.842, and 0.853 in the training cohort and 0.887 (0.842–0.923), 0.922, 0.799, and 0.853 in the testing cohort). STC2 alone also yielded acceptable results for early CRC detection (0.815 (0.776–0.849), 0.767, 0.849, and 0.829 in the training cohort and 0.870 (0.812–0.914), 0.952, 0.799, and 0.833 in the testing cohort). Moreover, STC2 maintained diagnostic accuracy for CRC patients with negative CEA (0.874 (0.842–0.901), 0.862, 0.849, and 0.853 in the training cohort and 0.898 (0.848–0.936), 0.930, 0.801, and 0.842 in the testing cohort). In comparison, the performances of the CEA and CA199 based Model_1 were far from satisfactory, especially in early cases (0.767 (0.726–0.805), 0.491, 0.863, and 0.771 in the training cohort and 0.817 (0.754–0.870), 0.476, 0.889, and 0.796 in the testing cohort).

**Conclusions:**

STC2 was a promising serum biomarker for CRC diagnosis either used alone or in combination with CXCL5 and CHI3L1.

## 1. Introduction

Colorectal cancer (CRC) is the third most commonly diagnosed cancer and the second leading cause of cancer death. There were approximately 1.93 million (10% of total cancer incidence) new cases of CRC and 0.94 million deaths (9.4% of total cancer death) in 2020, according to the latest statistics [1]. Moreover, the burden of CRC is expected to increase with 2.2 million new cases and 1.1 million deaths expected globally by 2030 [2]. The 5-year survival rate of CRC at late-stage is only 8%; however, with early intervention, it would dramatically increase to 90% [3]. Therefore, the most effective way to improve the patients' outcomes is early detection, which remains a challenge due to the late presentation of symptoms. Colonoscopy is currently considered as the gold standard for CRC diagnosis when combined with pathological examinations; however, the inconvenient preparation process, invasive procedure, and multiple complications lead to poor compliance and potential risks to patients [4]. For screening purpose, an ideal CRC test should be reliable and convenient. Serum biomarker is a noninvasive approach with high compliance. CEA is the most commonly used tumor biomarker; however, it is not recommended for screening, especially for early CRC because of low sensitivity and lack of CRC specificity [5]. The emerging epigenetic biomarkers [6], such as methylated septin9 and SDC2 (syndecan-2) genes, have been entered into clinical practice with a considerable improvement of diagnostic accuracy [7, 8]. The disadvantages of nuclear acid form of biomarkers are, nevertheless, obvious. Generally, measurement of nuclear acids in cell-free settings requires complicated pretreatment processes (extraction, purification, and bisulfite conversion), which lacks a robust automation platform. Meanwhile, the cost of these biomarkers is much higher than that of protein-based tests, limiting their use as a screening tool, especially in economically undeveloped regions. Therefore, it is urgently needed to develop novel protein biomarkers that are most applicable in clinical applications.

In the current study, three protein candidates, CXCL5 (C-X-C motif chemokine ligand 5), STC2 (stanniocalcin 2), and CHI3L1 (chitinase 3 like 1), were selected by analyzing the gene array datasets concerning CRC in GEO (Gene Expression Omnibus). CXCL5, also known as neutrophil activating peptide 78 (ENA-78), belongs to the CXC-type chemokine family. The sources of CXCL5 may be associated with cancer cell autocrine and paracrine loop in the tumor microenvironment (TME), transmitting signals by binding to the IL-8B receptor (CXCR2) [9]. STC2 is secreted as a phosphoprotein, and dysregulation of STC2 expression is linked with tumor progression and metastasis [10]. CHI3L1 is an indispensable member of the glycoside hydrolase family 18. Secreted by a multitude of cells including macrophages, neutrophils, as well as tumor cells, CHI3L1 plays a vital role in tissue injury, inflammation, tissue repair, and remodeling response. Increased serum CHI3L1 levels have been found to be involved in tumor development and progression. Consequently, CHI3L1 has been increasingly proposed as a sensitive biomarker and an attractive therapeutic target [11]. We compared the serum concentrations of these three candidate biomarkers under different settings and constructed a combined diagnostic model to evaluate the performance in CRC detection as well. The results demonstrated that the combination of three biomarkers was a powerful CRC diagnostic tool either for the whole group or early cases.

## 2. Materials and Methods

### 2.1. Potential Biomarker Selection

Three GEO datasets relating CRC gene expression (GSE44861 [12], GSE41258 [13], and GSE71187 [14]) were analyzed using the intrinsic R program of the GEO database. 30 shared upregulated genes (logFC >1 and adjusted *P* < 0.05) were determined (Supporting Figure 1). After searching GeneCards database for the subcellular location of their protein products (https://www.genecards.org), 16 genes with the highest confidence score for the extracellular were identified. We tested 9 candidates with commercially available kits (SPP1, MMP1, MMP3, EREG, LOXL2, THBS2, CXCL5, STC2, and CHI3L1) in a small portion of samples (40 subjects, 20 controls, and 20 CRCs); finally, CXCL5, STC2, and CHI3L1 with both detectable ELISA signals and significant level differences were selected for further measurement in the total samples (Supporting Table 1).

### 2.2. Study Population

The consecutive patients with newly diagnosed CRC (392) and polyp (342) were recruited from Jun 2020 to Sep 2021. All the subjects were diagnosed by colonoscopy and subsequently were confirmed by pathological examinations. The clinical stage of CRC of all patients was classified according to the TNM classification system of the American Joint Committee on Cancer [15]. CRC of stage 0 and Ι was considered as early-stage CRC. The underlying conditions (hypertension and diabetes) were judged based on corresponding standards. Infections were defined as the presence of one or more following conditions: suppurative peritonitis, acute pneumonia, and bacteremia but not included chronic infection with the virus, such as HBV or HCV. 153 subjects who visited the physical examination center of our hospital for checkups and exhibited no evidence of tumor or polyp were recruited as healthy controls. The characteristics of subjects were listed in Supporting Table 2; for 392 CRC patients, additional clinical information was summarized in Supporting Figure 2. Among the CRC subjects, 31 postsurgery serum samples were also collected when the patients were attending the hospital for their first time follow-up examination, usually six months after surgery. The experiments were performed in accordance with the regulation of the Institutional Ethics Committee of the Chengdu First People's Hospital.

### 2.3. Sample Collection and Assay

Blood samples were collected before receiving any chemotherapy or radiation therapy. The sera were isolated by centrifuge at 4000 rpm for 10 min and stored at −80° until use. CEA and CA199 were determined by electrochemiluminescence using the Cobas 8000 e602 analyzer (Roche Diagnostics, Germany). All ELISA kits were purchased from Boster Biological Technology (Wuhan, China) according to the manufacturer's instructions.

### 2.4. Statistical Analysis

Quantitative variables were first subjected to normality distribution and homogeneity of variances tests. Differences of serum levels were analyzed by one-way ANOVA (for more than 2 groups) or Student's *t-*test (for 2 groups) if the variables passed the assessment. If not, the Kruskal–Wallis test (for more than 2 groups) or the Mann–Whitney *U* test (for 2 groups) were used. Categorical variables were expressed as frequency (percentage) and analyzed using the chi-squared test. The whole data were then partitioned into training and testing cohorts at the ratio of 7 : 3 (627 in the training and 260 in the testing). Two combination models (Model_1 was the combination model of currently used biomarkers, CEA and CA-199, while Model_2 was the combination of 3 novel biomarkers, CXCL5, STC2, and CHI3L1) were constructed by binary logistic regression using the training cohort (variable details shown in Supporting Table 3), followed by ROC analysis to define the optimum cutoff values for an individual indicator or combination models on the basis of the maximum Youden index. The sensitivity, specificity, and overall accuracy of each indicator were calculated at the cutoffs given by ROC analysis using the training set in the first place. All the tests were performed using IBM SPSS software v.23, except for the pairwise correlations between five indicators, which were performed using R software 4.1.2.

## 3. Results

**Figure 1:**
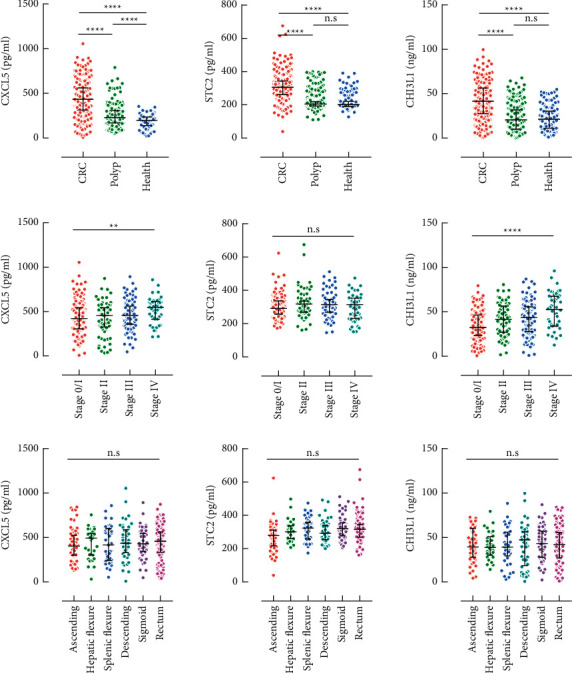
Comparison of serum concentrations of CXCL5, STC2, and CHI3L1 between three major groups (upper), CRC subpopulations by stage (middle), and CRC subpopulations by primary tumor location (lower). n.s, nonsignificant, ^*∗*^*P* < 0.05, ^*∗∗*^*P* < 0.01, and ^*∗∗∗∗*^*P* < 0.0001.

We first compared the serum levels of CXCL5, STC2, and CHI3L1 in three major groups. As predicted by the gene expressing profile, these proteins were all remarkably higher in CRC patients than in polyp patients or healthy controls. For STC2 and CHI3L1, no significant change was observed in polyp patients in comparison with healthy controls (Figure 1, upper). In the CRC group, levels of three indicators were also compared between stages and overall tendencies of elevation were found for CXCL5 and CHI3L1 but not for STC2, along with the disease progress (Figure 1, middle). Next, we compared them in the CRC group divided by primary tumor locations, for tumors in the proximal colon (right side, including the ascending colon and hepatic flexure) and the distal colon (left side, including the splenic flexure, descending colon, sigmoid, and rectum) exhibited different molecular characteristics and histology [16]. None of the three indicators showed a significant change between different sites (Figure 1, lower).

**Figure 2:**
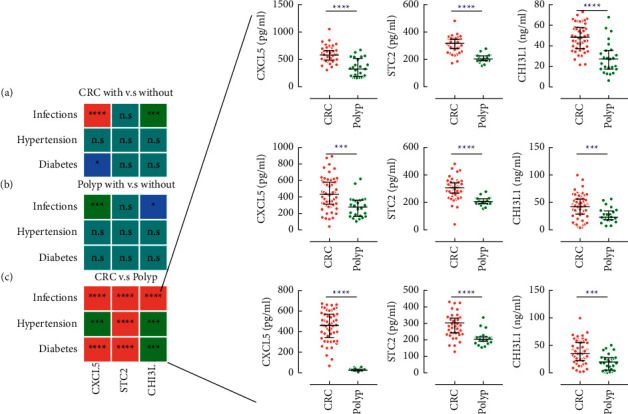
Statistic summary for comparison of CXCL5, STC2, and CHI3L1 levels, in CRC patients (a), polyp patients (b) with or without the indicating conditions, and between CRC and polyp patients with the indicating conditions (c). n.s, nonsignificant ^*∗*^*P* < 0.05, ^*∗∗∗*^*P* < 0.001, and^*∗∗∗∗*^*P* < 0.0001. Infections were defined as the presence of one or more following conditions: suppurative peritonitis, acute pneumonia, and bacteremia but not included chronic infection with virus, such as HBV or HCV.

In the polyp and CRC groups, some patients with underlying diseases, such as acute infections, hypertension, and diabetes, were diagnosed concurrently (detailed proportions found in Supporting Table 2). Concentration differences in the CRC or polyp groups with or without such diseases were compared, and the results are shown in Supporting Figures 3 and 4; in the main text, we presented the statistical summary in Figure 2. Intriguingly, STC2 levels did not differ significantly in both CRC and polyp patients with or without any kind of underlying diseases. However, CXCL5 and CHI3L1 were both increased in the subgroup of infection in both CRC and polyp patients (Figures 2(a) and 2(b), Supporting Figures 3 and 4). These results could be explained by the nature of CXCL5 and CHI3L1. CXCL5 has long been documented as one of the important chemokines that are expressed by many immune cells, such as macrophages, eosinophils, and cancer cells [17]. CHI3L1, which is also synthesized and secreted by a multitude of cells including macrophages and neutrophils, plays a major role in tissue injury, inflammation, tissue repair, and remodeling responses [18]. The higher levels of these two molecules in the sera of patients with infections might reflect the underlying inflammation response. The levels were further compared between CRC and polyp under each of these conditions. All three molecules were significantly higher in the tumor population (Figure 2(c)).

**Figure 3:**
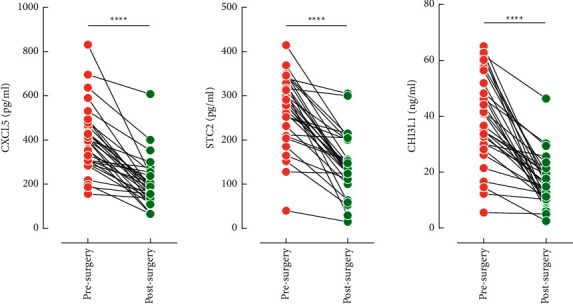
Serum levels of CXCL5, STC2, and CHI3L1 after surgery. ^*∗∗∗∗*^*P* < 0.0001.

Based on these findings, we concluded that tumor cells were the major source of three indicators in serum. Infections could promote their elevation, which may be further driven by cancer development. Among them, the STC2, whose serum level was not influenced by infections, may be the most promising marker for the diagnosis of CRC from benign diseases. To further explore the tumor-derived origin of these indicators, the sera of 31 postsurgery CRC patients were tested again. Compared with presurgery counterparts, the serum levels of the three indicators were all sharply dropped (Figure 3), further indicating that the increased amounts were mainly from tumor tissues. Pairwise correlations were also performed among them and CEA, CA199, the highest coefficient of 0.369 between CXCL5 and STC2, revealed overall weak correlations (Supporting Figure 5).

**Figure 4:**
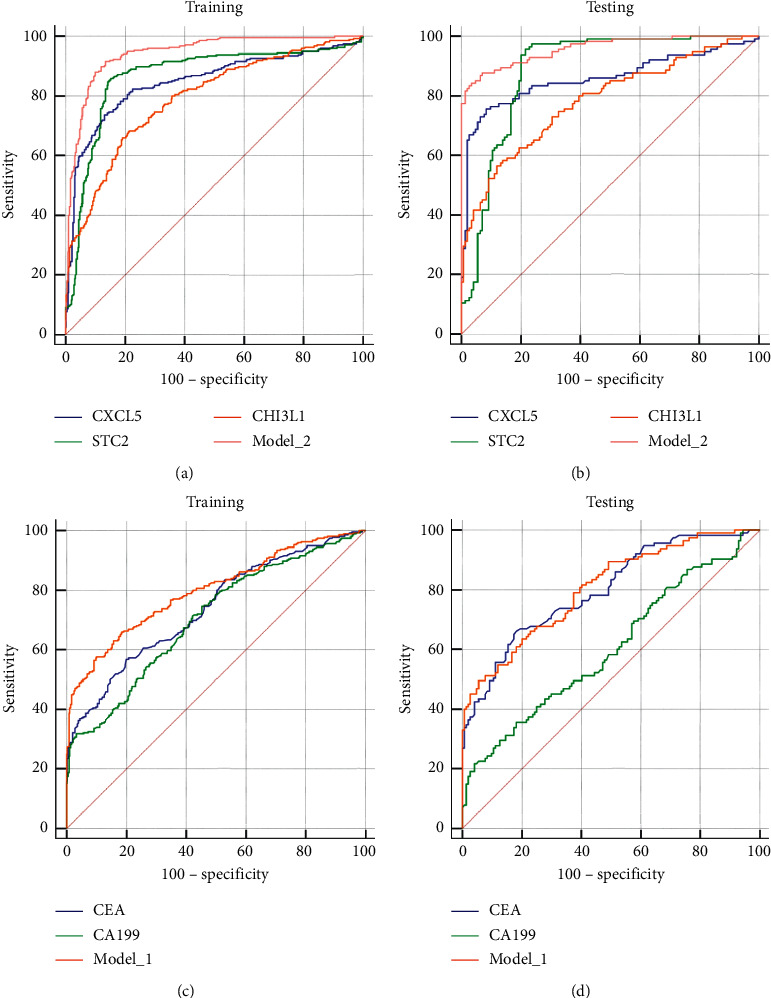
ROC curves of indicators in detection of CRC from all controls. CXCL5, STC2, CHI3L1, and their combination in the training (a) or testing (b) set. CEA, CA199, and their combination in the training (c) or testing (d) set.

We next evaluated the diagnostic power of CXCL5, STC2, CHI3L1, and their combination (Model_2), and the power of currently used CEA, CA199, and their combination (Model_1) was investigated as well. The ROC curve (Figure 4(a)) revealed Model_2 as the most powerful approach for discriminating CRC from non-CRC controls (polyp and health). The optimum cutoff value for Model_2 was 0.517 (AUC, 0.943 (95% CI 0.922–0.960); sensitivity, 0.848; specificity, 0.917; and accuracy, 0.886) (Table 1). When used as a single indicator, STC2 showed the best performance at the cutoff of 232.4 pg/ml (AUC, 0.866 (95% CI 0.837–0.892); sensitivity, 0.859; specificity, 0.842; and accuracy, 0.853) (Figure 4(a), Table 1). Similar results were found in the testing set, and for Model_2 at the same cutoff, the AUC, sensitivity, specificity, and accuracy were 0.959 (95% CI 0.927–0.980), 0.878, 0.917, and 0.900, respectively (Figure 4(b), Table 1). The performances of CEA, CA199, and their combined Model_1 are also listed in Table 1. The ROC curves for training and testing sets are shown in Figures 4(c) and 4(d), respectively. The optimum cutoff values for CEA and CA199 were 4.44 ng/ml and 37.3 U/ml, slightly lower than the recommended clinical cutoff of 5 ng/ml and 39 U/ml, respectively. Although the combination with CA199 (Model_1) enhanced the diagnostic power of CEA in both training and testing cohorts at the cutoff of 0.5194, the overall accuracy was considerably lower than that of Model_2 (0.753 vs. 0.887 in the training set and 0.726 vs. 0.900 in the testing set).

**Figure 5:**
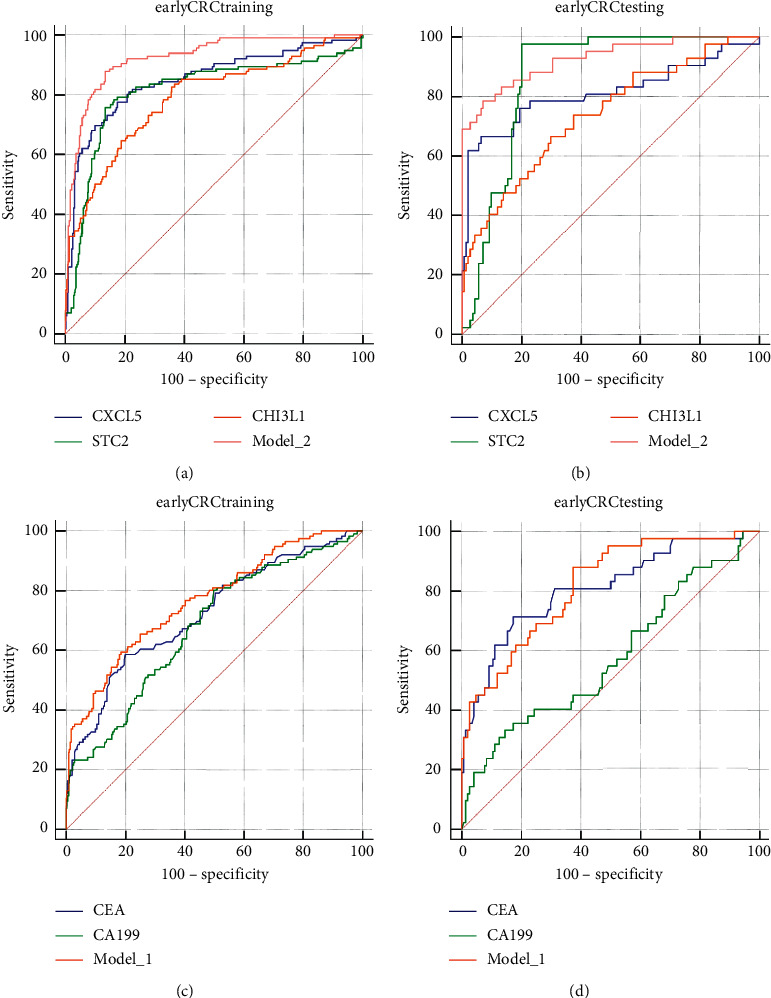
ROC curves of indicators in detection of early CRC from all controls. CXCL5, STC2, CHI3L1, and their combination in the training (a) or testing (b) set. CEA, CA199, and their combination in training (c) or testing (d) set.

The performances in detecting early CRC were determined next. ROC curves showed that Model_2 was still the most efficient approach among the six (Table 2) in both training and testing cohorts (Figures 5(a) and 5(b)). The STC2 was the suboptimum indicator which revealed AUC, sensitivity, specificity, and accuracy of 0.815 (95% CI 0.776–0.849), 0.767, 0.849, and 0.829, respectively, in the training cohort and those of 0.870 (95 CI 0.812–0.914), 0.952, 0.799, and 0.833 in the testing cohort. These results vastly outperformed the widely used CEA with those of 0.724 (95% CI 0.681–0.764), 0.431, 0.869, and 0.780 in the training cohort and 0.810 (95% CI 0.747–0.864), 0.476, 0.909, and 0.715 in the testing cohort. The outcome of CEA was slightly improved in combination with CA199, yet the sensitivities in both cohorts were still below 0.5 (Table 2, Figures 5(c) and 5(d)).

**Figure 6:**
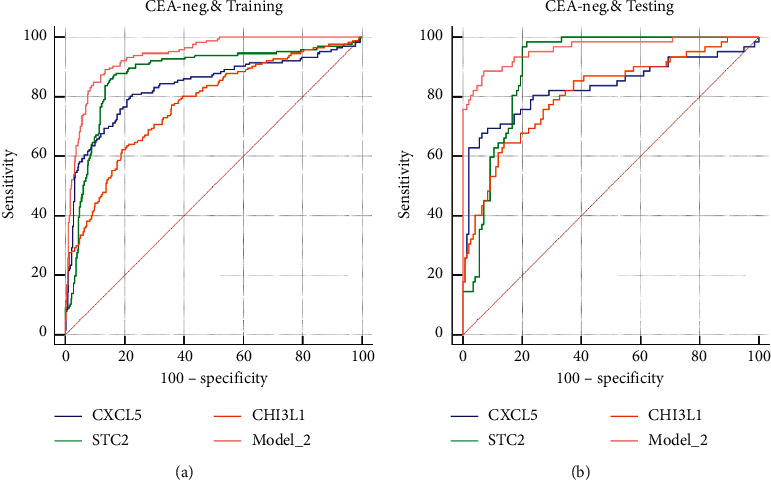
ROC curves of indicators in detection of CEA-negative CRC in the training (a) or testing (b) set.

The baseline characteristics revealed that only 163 cases (46.3%) were tested CEA positive among the CRC population and judged by the clinically used cutoff of 5 ng/ml. For the screening purpose, a reliable biomarker was necessary to distinguish the CEA-negative CRC from benign disease or healthy population. Therefore, we finally investigated the efficiency of the three novel candidate biomarkers in the detection of the CEA-negative CRC. For Model_2, AUC of 0.938 (95% CI 0.913–0.957) and 0.961 (95% CI 0.925–0.983) was achieved in the training and testing cohort, respectively (sensitivity, 0.826; specificity, 0.917; and accuracy, 0.888, in the training cohort and 0.887, 0.931, and 0.918 in the testing cohort). STC2 still was the best indicator among the three when used alone (AUC, 0.874 (95% CI 0.842–0.901); sensitivity, 0.862; specificity, 0.849; and accuracy, 0.853, in the training cohort and 0.898 (95% CI 0.848–0.936), 0.930, 0.801, and 0.842 in the testing cohort) (Table 3, Figures 6(a) and 6(b)).

## 4. Discussion

The development of new approaches based on serological biomarkers is an important goal in cancer diagnosis, especially for early screening [19]. Currently, the widely used biomarker for gastrointestinal tract cancer, CEA, was first isolated in 1956 [20]; after decades of clinical practice, low sensitivity and specificity (30–40% and 87%, respectively) have largely limited its application in early diagnosis of CRC [5, 21]. Thus, this biomarker is more suitable for detecting the recurrence of cancer following surgical/medical treatment [22, 23]. In the present data, CEA only yielded a sensitivity of 0.569 or 0.431 at the cutoff of 4.44 ng/ml in the detection of CRC or early CRC, respectively, which was far from satisfactory. In combination with another tumor marker, CA199 did not improve the diagnostic efficiency obviously, with 0.576 for the total CRC and 0.491 for the early CRC, respectively. These results are consistent with the recent finding [24], in which 0.543 of sensitivity was achieved by CAE and CA199 together for total CRC detection. In recent years, nuclear acid biomarkers have emerged as the more powerful tools for noninvasive surveillance of CRC. For instance, the methylation of Septin9 (^m^SEPT9) gene and its application in CRC patients' management have been intensively studied in the past decade. The sensitivity and specificity of ^m^SEPT9 for CRC ranged from 0.69 to 0.95 and from 0.81 to 1, respectively [7]. Another promising epigenetic biomarker is the SDC2 (Syndecan-2) gene methylation in the stool. Sensitivities of ^m^SDC2 for stages I/II were between 83.3 and 91.4%, and 89.6 and 100% for stage III/IV [8]. Although these improvements (in comparison with CEA) were significant, two shortcomings would limit the clinical popularization and promotion. Foremost, the cost of epigenetic marker detection is at least ten times higher than that of CEA, whose measurement depended on immunological modalities. For early-stage patients (or high risk) having no complaint, high cost has greatly dampened their willingness, especially in developing counties. The second obstacle affecting its use is the methodology itself. Measurement of nuclear acid in cell-free specimen (plasma, urine, and stool) requires complicated pretreatments which are relying heavily on manual processing. Moreover, to guarantee the stability of cell-free nuclear acid, sample collection and storage also need high dependence on operator expertise. In these regards, protein markers measurable in serum, providing objective and reproducible results, are the most applicable for regular surveillance [25–27].

Three potential tumor markers of protein form were introduced in the current study. We compared their performances in the detection of CRC either in early-stage or presented CEA-negative results. Measurement of them together and the utility of the combination model were proven to be the most powerful strategy for all cases, while STC2 showed excellent performance as used individually, which might be associated with its level constancy in the infectious status. An ideal tumor biomarker should meet the criteria of specific overexpression in cancer cells [28]. A great portion of emerging protein biomarkers was concurrently characterized as acute response proteins (ARPs), which increased in inflammatory situations. For example, osteopontin (OPN/SPP1) that regulates host immunity [29] has long been documented as a diagnostic or prognostic biomarker for a variety of cancers [30]. Our results clearly revealed that serum concentrations of SCT2 were not affected by the baseline conditions, such as infections, hypertension, or diabetes that are frequently found in the older patients. Regulation of STC2 expression is connected with two essential conditions, namely, hypoxia and ER (endoplasmic reticulum) stress associated with the tumor microenvironment [31, 32]. Due to rapid protein turnover to ensure the unprogrammed growth, tumor cells are inclined to generate harmful unfolded/misfolded proteins, which, in turn, trigger a cytoprotective response named unfolded protein reaction (UPR) [33]. STC2 is upregulated by ATF4 (activating transcription factor 4), an ER-resident transcription factor, that translocated into the nucleus under UPR [31, 34]. Apart from its cytoprotective role under stress conditions, STC2 is correlated with tumor invasion, metastasis, and size [35]. In head and neck squamous cell carcinoma, STC2 promotes metastasis through modulating the PI3K/AKT/Snail signaling [36]. As a secretory protein, STC2 also receives a great deal of attention for its potential use as a serum biomarker. In CRC patients, high STC2 expression is positively correlated with shorter overall survival [37]. In high-grade serous cancer, STC2 expression is significantly associated with the tumor grade and histotype and indicative of unfavorable outcomes [38]. Herein, we reported STC2 as an efficient diagnostic biomarker for CRC that reached comparable efficiency to those of methylation genes, with a high degree of convenience. However, combination with the other two markers did not seem to notably improve the predictive performance of STC2 alone (0.859 vs. 0.848 in sensitivity and 0.842 vs. 0.917 in specificity). The performances of CXCL5 and CHI3L1 may be influenced by nonspecific elevations resulting from underlying conditions, which led to relatively higher cutoff values (317.05 pg/ml and 34.14 ng/ml, respectively). Higher cutoffs produced higher specificities along with lower sensitivities in our results. Besides, both CXCL5 and CHI3L1 levels were positively correlated with CRC stages, indicating unsatisfactory capacities in early CRC detection.

In conclusion, these data identified a reliable and applicable serological tool for CRC diagnosis.

## Figures and Tables

**Table 1:** Results for indicated biomarkers in the diagnosis of CRC from the non-CRC controls.

	Training set (*n* = 627, 276 CRCs vs. 351 controls)				Testing set (*n* = 260, 116 CRCs vs. 144 controls)			
	AUC (95% CI)	Sensitivity	Specificity	Accuracy	AUC (95% CI)	Sensitivity	Specificity	Accuracy
CEA	0.739 (0.703–0.773)	0.569	0.801	0.699	0.799 (0.745–0.864)	0.661	0.819	0.749
CA 199	0.706 (0.668–0.741)	0.747	0.547	0.635	0.602 (0.539–0.662)	0.357	0.819	0.614
Model_1	0.798 (0.765–0.829)	0.576	0.903	0.753	0.804 (0.750–0.851)	0.591	0.833	0.726
CXCL5	0.847 (0.817–0.874)	0.736	0.869	0.811	0.859 (0.811–0.899)	0.774	0.861	0.846
STC2	0.866 (0.837–0.892)	0.859	0.842	0.853	0.887 (0.842–0.923)	0.922	0.799	0.853
CHI3L1	0.792 (0.758–0.823)	0.661	0.809	0.744	0.783 (0.728–0.831)	0.591	0.840	0.729
Model_2	0.943 (0.922–0.960)	0.848	0.917	0.887	0.959 (0.927–0.980)	0.878	0.917	0.900

Cutoffs: CEA: 4.44 ng/ml, CA199: 37.3 U/ml, CXCL5: 317.05 pg/ml, STC2: 232.4 pg/ml, CHI3L1: 34.41 ng/ml, Model_1: 0.5194, and Model_2: 0.517. The cutoffs were given according to the maximum Youden index calculated by ROC using training data and were then applied in the following analysis to obtain the corresponding parameters (sensitivity, specificity, and accuracy).

**Table 2:** Results for indicated biomarkers in the diagnosis of early CRC from the non-CRC controls.

	Training set (*n* = 467, 116 CRCs vs. 351 controls)				Testing set (*n* = 186,42 CRCs vs. 144 controls)			
	AUC (95% CI)	Sensitivity	Specificity	Accuracy	AUC (95% CI)	Sensitivity	Specificity	Accuracy
CEA	0.724 (0.681–0.764)	0.431	0.869	0.780	0.810 (0.747–0.864)	0.476	0.909	0.715
CA199	0.677 (0.633–0.720)	0.353	0.798	0.687	0.577 (0.503–0.649)	0.357	0.819	0.715
Model_1	0.767 (0.726–0.805)	0.491	0.863	0.771	0.817 (0.754–0.870)	0.476	0.889	0.796
CXCL5	0.851 (0.815–0.892)	0.776	0.868	0.845	0.812 (0.749–0.866)	0.667	0.861	0.817
STC2	0.815 (0.776–0.849)	0.767	0.849	0.829	0.870 (0.812–0.914)	0.952	0.799	0.833
CHI3L1	0.790 (0.750–0.826)	0.647	0.809	0.769	0.738 (0.669–0.800)	0.476	0.840	0.758
Model_2	0.925 (0.897–0.947)	0.793	0.917	0.886	0.926 (0.979–0.959)	0.786	0.931	0.898

Early CRC was defined as stage 0 and Ι.

**Table 3:** Results for indicated biomarkers in the diagnosis of CEA-negative CRC from the non-CRC controls.

	Training set (*n* = 518, 167 CRCs vs. 351 controls)				Testing set (*n* = 206, 62 CRCs vs. 144 controls)			
	AUC (95% CI)	Sensitivity	Specificity	Accuracy	AUC (95% CI)	Sensitivity	Specificity	Accuracy
CXCL5	0.834 (0.799–0.865)	0.695	0.869	0.813	0.834 (0.776–0.882)	0.710	0.861	0.816
STC2	0.874 (0.842–0.901)	0.862	0.849	0.853	0.898 (0.848–0.936)	0.930	0.801	0.842
CHI3L1	0.772 (0.734–0.808)	0.623	0.809	0.749	0.810 (0.750–0.862)	0.645	0.840	0.781
Model_2	0.938 (0.913–0.957)	0.826	0.917	0.888	0.961 (0.925–0.983)	0.887	0.931	0.918

## Data Availability

The data that support the findings of this study are available from the corresponding author upon reasonable request.

## Supplementary Materials

Supplementary Materials Brief description of supporting materials. *Supporting Table 1.* Upregulated genes shared by three GEO datasets. *Supporting Table 2*. Baseline characteristics of enrolled cases. *Supporting Table 3*. Variables in three combination models based on the training set (*n* = 627, 70% of the total case). *Supporting Figure 1*. Brief information of 3 GEO dataset and the numbers of shared upregulated genes. *Supporting Figure 2*. Clinical features of 392 CRC patients. *Supporting Figure 3*. Comparison of CXCL5, STC2, and CHI3L1 serum levels in CRC patients with or without infections (upper), hypertension (middle), and diabetes (lower). *Supporting Figure 4*. Comparison of CXCL5, STC2, and CHI3L1 serum levels in polyp patients with or without infections (upper), hypertension (middle), and diabetes (lower). *Supporting Figure 5*. Correlations of levels of 5 indicating biomarkers.
