# Supplementary material for: Combined Efficacy of CXCL5, STC2, and CHI3L1 in the Diagnosis of Colorectal Cancer
Source: J Oncol. 2022 May 20;2022:7271514. doi: 10.1155/2022/7271514 (PMC9142324; doi:10.1155/2022/7271514)
Supplement: Supplementary Materials — Brief description of supporting materials. Supporting Table 1. Upregulated genes shared by three GEO datasets. Supporting Table 2. Baseline characteristics of enrolled cases. Supporting Table 3. Variables in three combination models based on the training set (n = 627, 70% of the total case). Supporting Figure 1. Brief information of 3 GEO dataset and the numbers of shared upregulated genes. Supporting Figure 2. Clinical features of 392 CRC patients. Supporting Figure 3. Comparison of CXCL5, STC2, and CHI3L1 serum levels in CRC patients with or without infections (upper), hypertension (middle), and diabetes (lower). Supporting Figure 4. Comparison of CXCL5, STC2, and CHI3L1 serum levels in polyp patients with or without infections (upper), hypertension (middle), and diabetes (lower). Supporting Figure 5. Correlations of levels of 5 indicating biomarkers. [file 7271514.f1.pdf]

Brief description

- Supporting table 1. Upregulated genes shared by three GEO datasets.
- Supporting table 2. Baseline characteristics of enrolled cases.
- Supporting table 3. Variables in three combination models based on the training set (n= 627, 70% of the total case).
- Supporting figure 1. Brief information of 3 GEO dataset and the numbers of shared upregulated genes.
- Supporting figure 2. Clinical features of 392 CRC patients.
- Supporting figure 3 . Comparison of CXCL5, STC2, and CHI3L1 serum levels in CRC patients with or without infections (upper), hypertension (middle) and diabetes (lower).
- Supporting figure 4. Comparison of CXCL5, STC2, and CHI3L1 serum levels in polyp patients with or without infections (upper), hypertension (middle) and diabetes (lower).
- Supporting figure 5. Correlations of levels of 5 indicating biomarkers.

Supporting table 1. Upregulated genes shared by three GEO datasets

Symbol	Title	Location	Log Fold change		
			GSE44861	GSE41258	GSE71187
CDH3	cadherin 3	3	1.713	3.458	1.113
**CHI3L1**	**chitinase 3 like 1**	5	1.500	3.178	1.743
COL11A1	collagen type XI alpha 1 chain	5	2.098	4.05	1.456
COL1A1	collagen type I alpha 1 chain	5	1.612	2.599	1.715
COL1A2	collagen type I alpha 1 chain	5	1..667	2.308	1.177
COL4A1	collagen type IV alpha 1 chain	5	1.474	1.216	1.312
COL5A1	collagen type V alpha 1 chain	5	1.154	1.513	1.180
COL5A2	collagen type V alpha 2 chain	5	1.293	1.581	1.310
**CXCL5**	**C-X-C motif chemokine ligand 5**	5	2.100	1.489	1.412
DPEP1	dipeptidase 1	4	1.623	1.846	2.149
EDNRA	endothelin receptor type A	2	1.169	1.824	1.063
ELOVL5	ELOVL fatty acid elongase 5	2	1.124	1.038	1.566
EREG	epiregulin	5	1.637	1.854	2.068
FAP	fibroblast activation protein alpha	5	1.577	2.625	1.767
INHBA	inhibin beta A subunit	5	2.230	2.817	1.714
KRT23	keratin 23	1	2.290	1.965	1.839
LOXL2	lysyl oxidase like 2	5	1.069	1.136	1.141
MFAP2	microfibrillar associated protein 2	5	1.157	2.219	1.111
MMP1	matrix metallopeptidase 1	5	1.797	2.089	1.431
MMP3	matrix metallopeptidase 3	5	1.598	1.296	2.104
SCD	stearoyl-CoA desaturase	3	1.408	1.215	1.071
SLC7A5	solute carrier family 7 member 11	4	1.196	1.327	1.778
SLCO4A1	solute carrier organic anion transporter member 4A1	1	1.117	1.204	1.037
SPARC	secreted protein acidic and cysteine rich	5	1.098	1.549	1.279
SPP1	secreted phosphoprotein 1	5	2.280	2.839	1.707
**STC2**	**stanniocalcin 2**	5	1.081	1.079	1.754
SULF1	sulfatase 1	5	1.599	2.126	1.136
THBS2	thrombospondin 2	5	1.590	2.741	1.483
TPX2	TPX2, microtubule nucleation factor	4	1.200	1.112	1.071
TRIB3	tribbles pseudokinase 3	2	1.462	1.553	1.173

Digits in the location column represent the confidence scores of extracellular location of the gene products, range (0-5), data were extracted from genecards base (https://www.genecards.org). Genes marked in bold were explored in the current study.

Supporting table 2. Baseline characteristics of enrolled cases

	Health (n=153)	Polyp (n=342)	CRC (n=392)	P
Age (Year)	45 [41-58]	51.5 [43.25-68.75]	64 [49.25-69]	**
Sex (Male, %)	52.56%	53.89%	55.27%	n.s
Infections (n, %)		25, (7.3%)	45, (11.5%)	**
Hypertension (n, %)		24, (7.0%)	63, (16.1%)	***
Diabetes (n, %)		28 (8.2%)	54, (13.8%)	**
CEA (ng/ml)	2.53 [1.53-3.42]	2.3 [1.43-3.25]	3.68 [2.55-8.68]	****
CA199 (U/ml)	14.32 [5.4-21.36]	11.73 [5.05-21.73]	20.46 [10.54-46.48]	****
CEA positive (n, %)	12 (7.8 %)	29 (8.5%)	163 (41.6%)	****
CA199 positive (n, %)	6 (3.9%)	27 (8.0%)	117 (29.8%)	****

Data in square brackets were described as median with quantile (Q1-Q3). n.s non-significant, *P<0.05, **P<0.01, ***P<0.001, ****P<0.0001

Supporting table 3. Variables in three combination models based on the training set (n= 627, 70% of the total case)

		B	S.E	Wald	DF	Sig.	Exp(B)	95% CI lower	95% CI upper
Model_1	CEA	0.372	0.051	52.639	1	0.000	1.451	1.358	1.492
	CA199	0.040	0.006	47.768	1	0.000	1.041	1.024	1.067
	Contant	-2.493	.226	121.275	1	0.000	.083		
Model_2	CXCL5	0.008	0.001	71.005	1	0.000	1.008	1.006	1.010
	STC2	0.020	0.002	76.052	1	0.000	1.021	1.016	1.025
	CHI3L1	0.058	0.008	51.500	1	0.000	1.060	1.043	1.077
	Contant	-9.786	0.744	172.821	1	0.000	0.000		

All the models were constructed using training cohort that split from the total of 887 cases at the ratio of 0.7

Accession No.	Platform	Sample size	Upregulated# gene number
GSE41258	GPL96	390	155
GSE44861	GPL3921	111	139
GSE71187	GPL10558	284	229

log FC>1 and adj. P<0.05

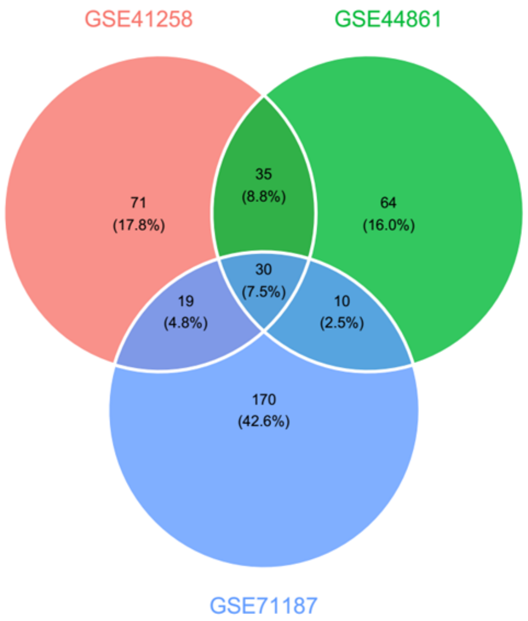

Supporting figure 1. Brief information of 3 GEO dataset and the numbers of shared upregulated genes.

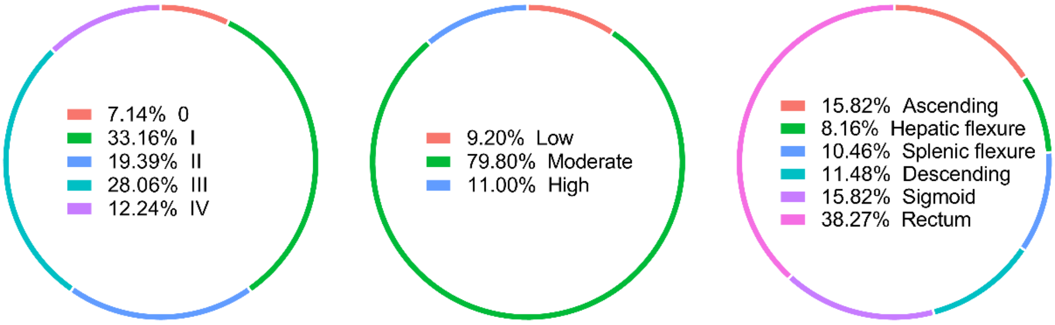

Supporting figure 2. Clinical features of 392 CRC patients

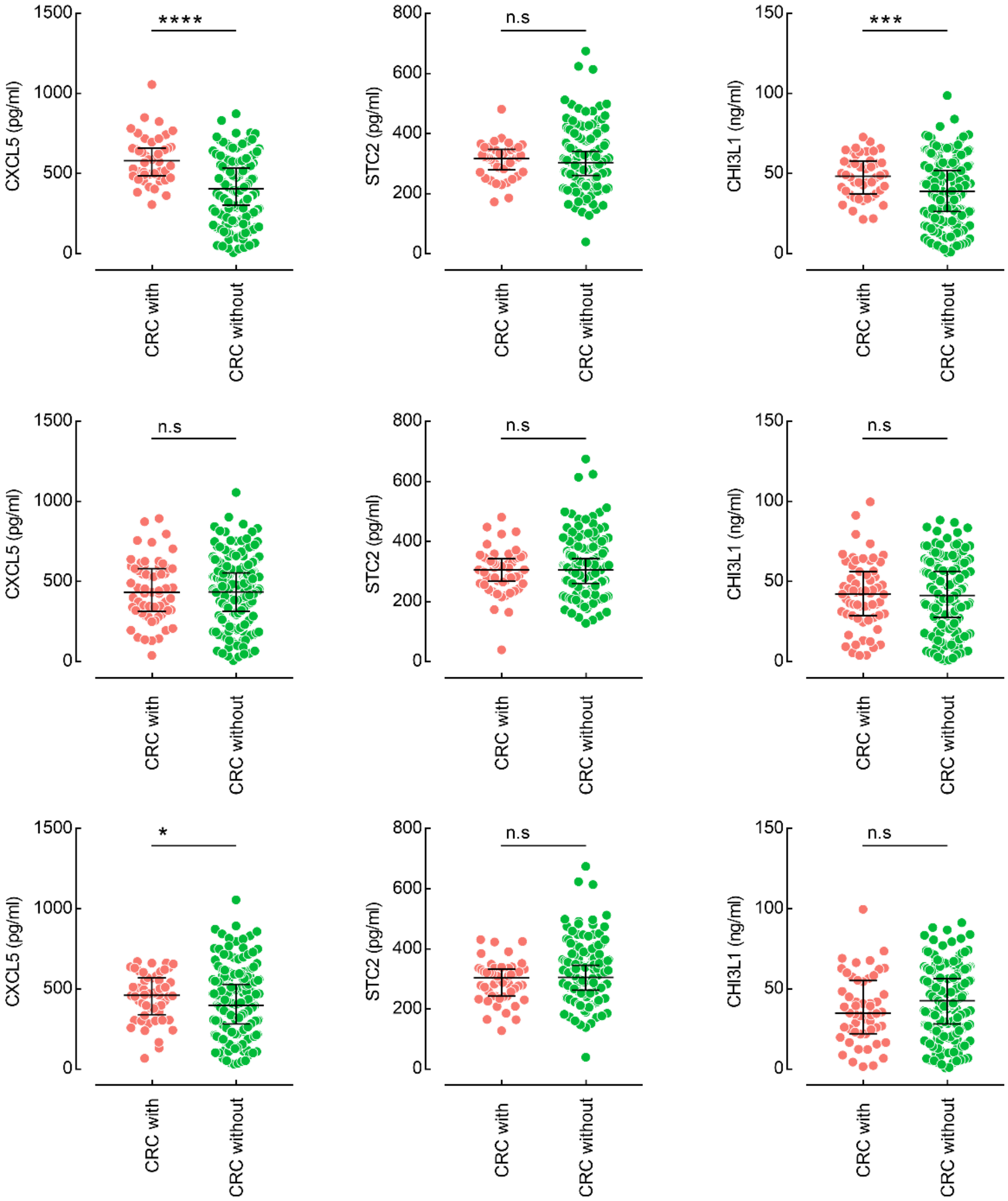

Supporting figure 3 . Comparison of CXCL5, STC2, and CHI3L1 serum levels in CRC patients with or without infections (upper), hypertension (middle) and diabetes (lower). * P<0.05, ***P< 0.001, **** P<0.0001, n.s non-significant.

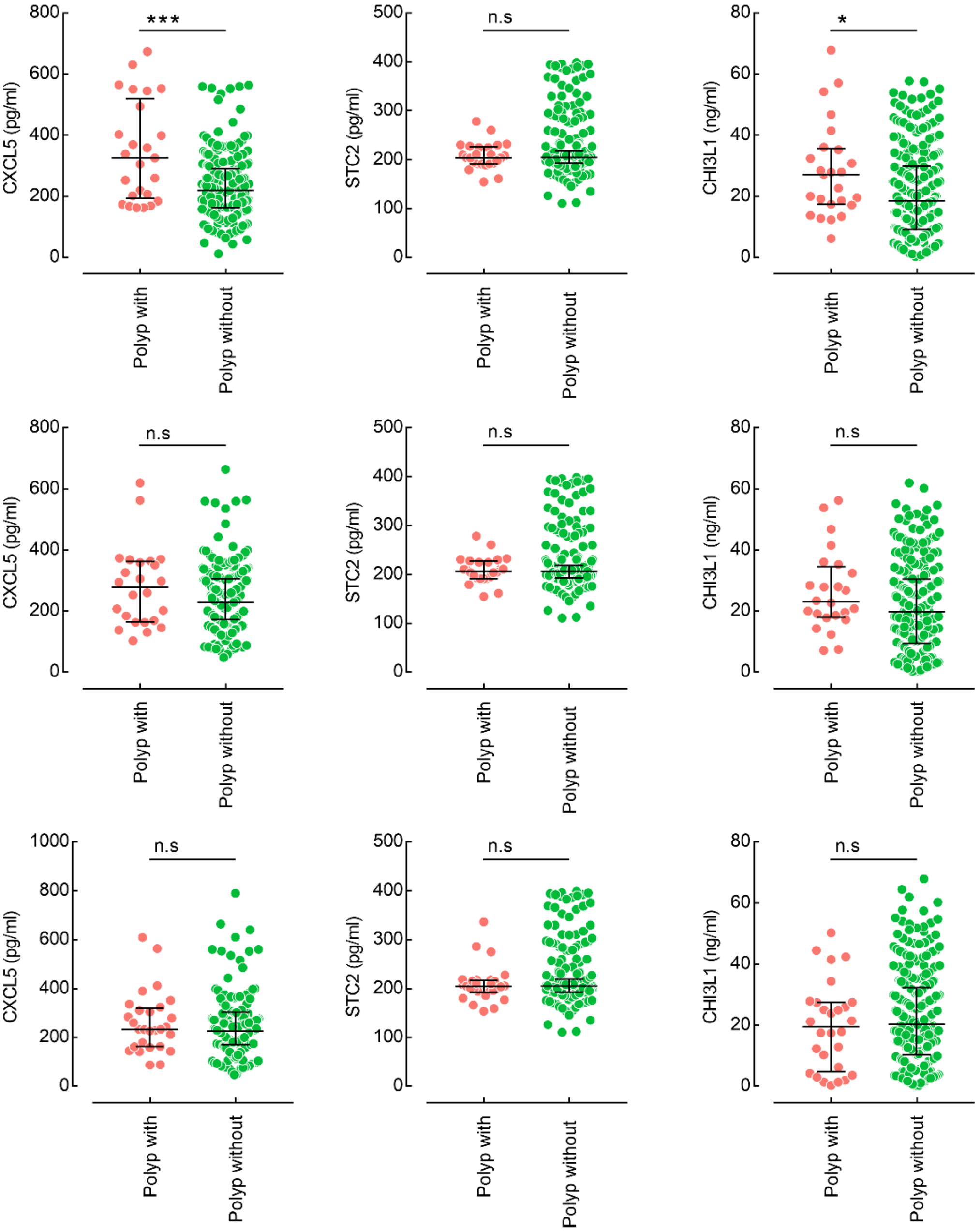

Supporting figure 4. Comparison of CXCL5, STC2, and CHI3L1 serum levels in polyp patients with or without infections (upper), hypertension (middle) and diabetes (lower). * P<0.05, ***P< 0.001, n.s non-significant.

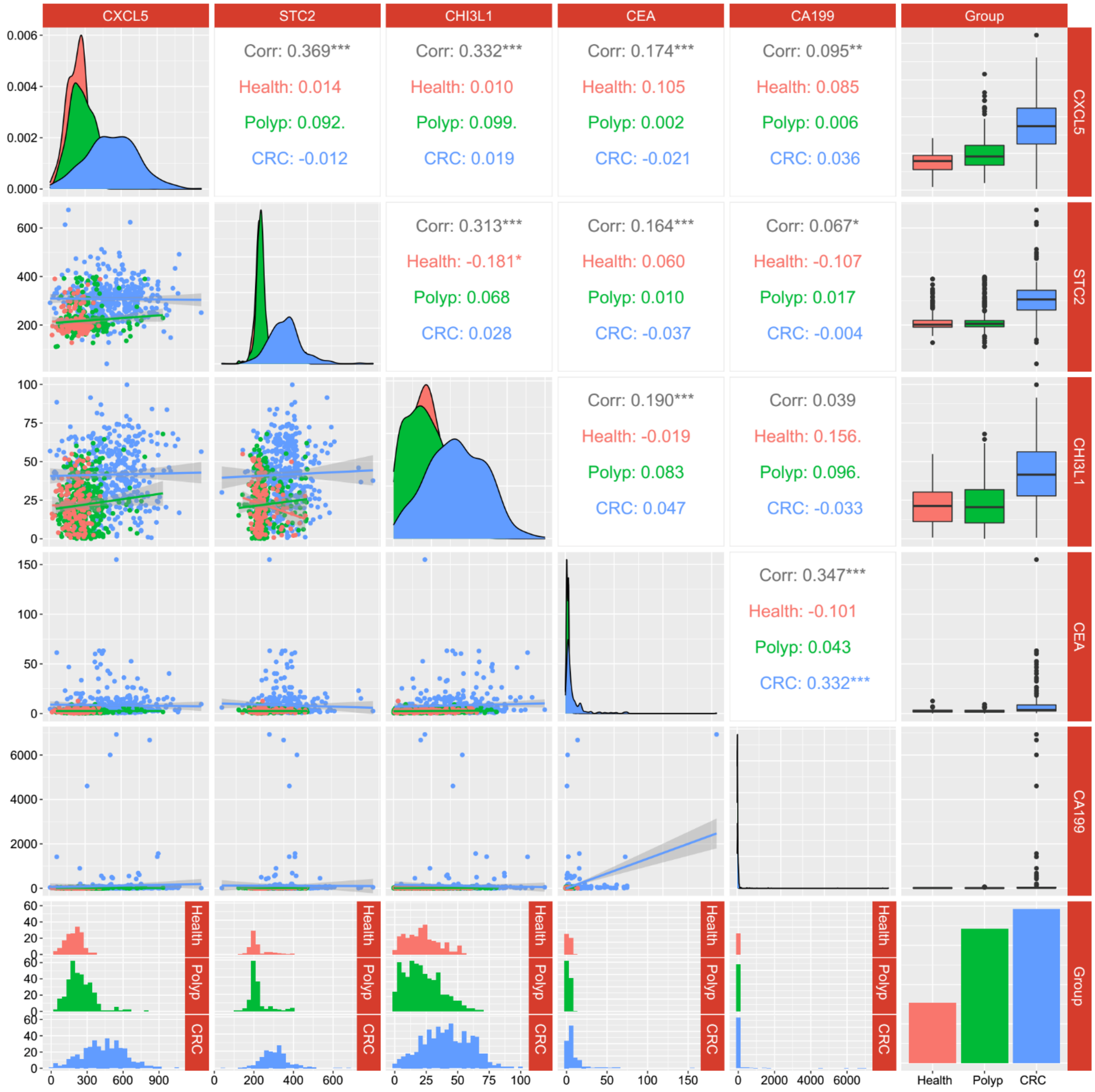

Supporting figure 5. Correlations of levels of 5 indicating biomarkers. Boxplots in in the right column showed the comparison of each biomarkers in three groups. The histograms in the lower panel showed the frequency distribution of their serum concentrations, which were also reflected by the kernel density plots at the diagonal. The dot plot in lower triangle presented the pairwise correlations. The Pearson correlation coefficients, showed in the upper triangle, indicated weak correlations between each biomarkers. Finally, the histogram in the lower right block represented the subject number of each group.
